# miRNA Signature in Mouse Spermatogonial Stem Cells Revealed by High-Throughput Sequencing

**DOI:** 10.1155/2014/154251

**Published:** 2014-07-20

**Authors:** Tao Tan, Yanfeng Zhang, Weizhi Ji, Ping Zheng

**Affiliations:** ^1^Yunnan Key Laboratory of Primate Biomedical Research, No. 1 Boda Road, Yuhua Area, Chenggong District, Kunming, Yunnan 650500, China; ^2^State Key Laboratory of Genetic Resources and Evolution, Kunming Institute of Zoology, Chinese Academy of Sciences, Kunming, Yunnan 650223, China; ^3^Kunming Biomed International and National Engineering Research Center of Biomedicine and Animal Science, Kunming, Yunnan 650500, China; ^4^Department of Medicine, School of Medicine, Vanderbilt University, Nashville, TN 37203, USA

## Abstract

Spermatogonial stem cells (SSCs) play fundamental roles in spermatogenesis. Although a handful of genes have been discovered as key regulators of SSC self-renewal and differentiation, the regulatory network responsible for SSC function remains unclear. In particular, small RNA signatures during mouse spermatogenesis are not yet systematically investigated. Here, using next generation sequencing, we compared small RNA signatures of in vitro expanded SSCs, testis-derived somatic cells (Sertoli cells), developing germ cells, mouse embryonic stem cells (ESCs), and mouse mesenchymal stem cells among mouse embryonic stem cells (ESCs) to address small RNA transition during mouse spermatogenesis. The results manifest that small RNA transition during mouse spermatogenesis displays overall declined expression profiles of miRNAs and endo-siRNAs, in parallel with elevated expression profiles of piRNAs, resulting in the normal biogenesis of sperms. Meanwhile, several novel miRNAs were preferentially expressed in mouse SSCs, and further investigation of their functional annotation will allow insights into the mechanisms involved in the regulation of SSC activities. We also demonstrated the similarity of miRNA signatures between SSCs and ESCs, thereby providing a new clue to understanding the molecular basis underlying the easy conversion of SSCs to ESCs.

## 1. Introduction

Embryonic development in mice involves the migration of primordial germ cells (GCs) to the genital ridge and their subsequent differentiation into gonocytes. At about 6 days after birth, the gonocytes in male mice either undergo a transition to spermatogonia stem cells (SSCs), the foundation for continuous spermatogenesis throughout the reproductive lifetime, or develop directly into type A1 spermatogonia [1]. Spermatogenesis does not occur until puberty (about 3 weeks after birth), at which time SSCs undergo active self-renewal and differentiation to give rise to daughter cells for spermatogenesis [1]. SSCs thus play a fundamental role in spermatogenesis and male reproductive biology. Abnormalities in SSC function and regulation are closely related to male infertility, and SSC transplantation has potential clinical applications. Furthermore, unipotent SSCs have unique features in terms of their capacity to be easily reprogrammed into pluripotent embryonic stem cell- (ESC-) like cells in culture. These SSC-derived pluripotent cells are generally referred to as germline-derived pluripotent stem cells (gPSCs). When seeded at low density (<8000 SSCs per well in 24-well plate), SSCs undergo spontaneous conversion into gPSCs without modification of the culture medium [2]. gPSCs can also be derived from neonatal or adult murine or human testicular tissue [3–8]. These gPSCs display morphological, functional, and molecular characteristics akin to ESCs [9, 10]. For example, they demonstrate pluripotent differentiation into cells forming all three germ layers and GCs [3, 4] and display similar gene, protein [11], and microRNA (miRNA) expression profiles [12], as well as epigenetic signatures, to ESCs. SSCs are therefore considered a potential source of pluripotent stem cells [13, 14].

The importance of SSCs means that numerous studies have investigated the regulation of self-renewal and differentiation activities of mouse and human SSCs in vivo or in vitro. Key genes, growth factors, and signaling pathways have been identified which are essential for SSC self-renewal and differentiation [15–22]. In addition, small noncoding RNAs also play essential roles in regulating SSC functions, such as spermatogenesis [23, 24]. Small RNAs are noncoding RNAs of 18–32 nt long, which can be further divided into three distinct classes: miRNAs, endogenous small interfering RNAs (endo-siRNAs), and Piwi-interacting RNAs (piRNAs). Comparisons of small noncoding RNA profiles in GCs at variable developmental stages and in testicular somatic cells identified a specific group of small noncoding RNAs important for SSC function. For instance, miR-34C is expressed specifically in mouse pachytene spermatocytes and in round spermatids and might play a role in regulating germ cell development [25]. miR-21 is highly expressed in mouse SSC populations and is important for self-renewal or homeostasis of SSCs [26].

Although SSCs are considered to be readily reprogrammed into gPSCs, the underlying mechanisms are poorly understood. Several pioneering studies have explored the possible mechanisms by comparing the molecular properties of SSCs and gPSCs (or ESCs). Kanatsu-Shinohara et al. examined the gene expression profiles of mouse SSCs and gPSCs by microarray analysis and revealed significant differences in mRNA expression patterns between these two cell types [27]. However, relatively fewer proteins were differentially expressed in gPSCs compared with SSCs in a proteomic assay [11]. Four transcription factors, including Oct4, Sox2, Klf4, and c-Myc, are widely used in reprogramming fibroblasts into pluripotent stem cells [28–30]. Although mRNAs of these Yamanaka factors were transcribed in SSCs, their expression levels were only 5–40% of those in pluripotent stem cells. Moreover, Sox2 protein expression was not detected and the protein levels of Oct4, Klf4, and c-Myc were extremely low in SSCs compared with ESCs or gPSCs [27]. Thus, the spontaneous conversion of SSCs to gPSCs cannot be explained simply by the rare transcription of reprogramming factors in SSCs. In this study, we investigated the whole-genome small noncoding RNA expression profiles of in vitro expanded mouse SSCs, developing GCs, mouse testis somatic cells (Sertoli cells (STs)), mouse ESCs, and mouse mesenchymal stem cells (MSCs). We compared their small noncoding RNA profiles and identified several highly expressed small RNAs in SSCs. Moreover, we found that ESCs and SSCs exhibited similar miRNA profiles, which could provide a new clue to understanding the molecular mechanisms underlying the spontaneous reprogramming of unipotent SSCs into multipotent gPSCs.

## 2. Materials and Methods

### 2.1. Ethics Statement

This study was carried out in strict accordance with the recommendations in the* Guide for the Care and Use of Laboratory Animals* of the National Research Council. The protocol was approved by the Institutional Animal Care and Use Committee (IACUC) of Kunming Institute of Zoology, Chinese Academy of Sciences. All surgery was performed under isoflurane anesthesia, and all efforts were made to minimize suffering.

### 2.2. Derivation and Expansion of Mouse SSCs

Testes were dissected from 4-5-week-old CD1 mice. Testicular tubules were isolated from the tunica albuginea and mechanically dissociated with forceps. The testicular tubules were then digested with 1 mg/mL collagenase IV for 15 min, washed twice with phosphate-buffered saline (PBS), and digested with 0.05% trypsin for 10 min. An equal volume of defined fetal bovine serum (FBS; Hyclone, Logan, UT, USA) was then added. The single-cell suspension was passed through a 30 *μ*m filter and centrifuged at 300 g for 5 min at room temperature, and the supernatant was aspirated. The cells were incubated with Feeder Removal MicroBeads (Miltenyi Biotec, Auburn, CA, USA) and the negatively-labeled cells were collected, according to the manufacturer's instructions. The cell suspension was then centrifuged at 300 g for 5 min at room temperature and the supernatant was aspirated. The cells were plated onto MEF feeders from E13.5 mouse fetuses (CD1) in SSC medium.

Mouse SSCs were cultured as described previously [31], with minor modifications. Briefly, SSCs were seeded onto MEF feeders from E13.5 mouse fetuses (CD1) and cultured in StemPro-34 (Invitrogen, Carlsbad, CA, USA) supplemented with 2 mM glutamine (Invitrogen), 0.1 mM mercaptoethanol, 1× nonessential amino acids (Invitrogen), 1× penicillin-streptomycin (Invitrogen), 1× sodium pyruvate (Invitrogen), 40 ng/mL glial cell-derived neurotrophic factor (R&D Systems, Minneapolis, MN, USA), 10 ng/mL epidermal growth factor, 10^3^ U/mL leukocyte migration inhibitory factor (Chemicon, Temecula, CA, USA), 10 ng/mL basic fibroblast growth factor (Chemicon), 60 *μ*M putrescine, and 10% defined FBS (Hyclone) (subsequently referred to as SSC medium). The cells were passaged with 0.05% trypsin every 6-7 days. All chemicals were from Sigma Chemical (St. Louis, MO, USA) unless otherwise stated.

### 2.3. Isolation and Expansion of MSCs

MSCs were generated from bone marrow from tibias and femurs of 4-5-week-old CD1 mice, as described previously [32]. Established MSCs were cultured in low-glucose DMEM medium supplemented with 10% defined FBS (Hyclone), 2 mM glutamine (Invitrogen), 100 U/mL penicillin (Invitrogen), and 100 mg/mL streptomycin (Invitrogen).

### 2.4. Sertoli Cells and Developing Germ Cells Purification

Sertoli cells and developing germ cells were isolated from 4-5-week-old CD1 mouse testicles as previously described [33]. Isolated cells were resuspended in 1 mL of Trizol (Invitrogen) for subsequent use.

### 2.5. Immunofluorescence and Confocal Microscopy

Cells were fixed with 4% paraformaldehyde for 10–15 min at 25°C and then rinsed three times in PBS, followed by permeabilization with 0.2% Triton X-100 for 10–15 min. Cells were then blocked in 5% goat serum for 30 min at 25°C and incubated with primary and secondary antibodies (Table  S1) before imaging under an LSM 510 META confocal microscope (Carl Zeiss, Jena, Germany) (see Supplementary Material available online at http://dx.doi.org/10.1155/2014/154251). Antibodies were obtained commercially and DNA was labeled with Hoechst 33342 or propidium iodide. An isotype-matched IgG was used as negative control in each experiment.

### 2.6. Reverse Transcription-Polymerase Chain Reaction

Total RNA was extracted from mouse MEF cells (negative control) and mouse SSCs using Trizol (Invitrogen). RNAs were subjected to treatment with DNase I (Fermentas, Vilnius, Lithuania) to remove possible genomic DNA contamination. Reverse transcription was carried out with approximately 2 *μ*g of total RNA using a RevertAid H Minus First strand cDNA synthesis kit (Fermentas). One microliter of RT products was added to 1× Reaction Ready HotStart PCR master mix (Takara, Dalian, China) in a final volume of 25 *μ*L and amplified under the following conditions: 1 cycle at 95°C for 5 min; 25–35 cycles at 95°C for 30 sec, 56–58°C for 30 sec, 72°C for 30 sec, and a full extension cycle at 72°C for 10 min. The sequences of the specific primer sets are provided in Table  S2. The polymerase chain reaction products were separated on 2% agarose gels and visualized after staining with ethidium bromide.

### 2.7. Flow Cytometric Analysis of Mouse Mesenchymal Stem Cell Surface Antigens

2 × 10^5^ mouse MSCs were harvested and incubated with 1 *μ*g of phycoerythrin (PE) conjugated antibodies or control isotype immunoglobulin Gs (IgGs) (Table  S1) at 4°C for 30 minutes. Samples were analyzed using a FACS vantage SE (BD Biosciences, San Jose, CA, USA).

### 2.8. RNA Extraction and Small RNA Sequencing

Total RNA was isolated from mouse ESCs, SSCs, GCs, STs, and MSCs using Trizol (Invitrogen). Ten micrograms of total RNA from each sample was separated by 15% denaturing polyacrylamide gel electrophoresis and visualized by SYBR-gold staining. Small RNAs of 18–40 nt were gel-purified, and cDNAs were prepared using the Illumina small RNA preparation kit (Illumina, San Diego, CA, USA) and sequenced using the Illumina HiSeq 2000.

### 2.9. High-Throughput Sequencing Analysis and Annotation of Small RNAs

The Illumina base-calling pipeline was used for fluorescent image deconvolution, quality value calculation, and sequence conversion to obtain reads with a length of 50 nt. High-quality (clean) reads were obtained after trimming the 5′ and 3′ adaptors and eliminating contaminants and inadequate (<18 nt) and low-quality reads. The clean reads were then mapped to the mouse genome (mm9) using SOAP2 [34]. Perfectly matched reads were summarized and retained for further analyses. Read annotations were performed as described previously. Briefly, a hierarchical order that classified reads into specific RNA species was determined for annotation using the BLASTn (ftp://ftp.ncbi.nih.gov/blast/) program. The annotation order was miRNA > rRNA/snoRNA/tRNA/scRNA/snRNA > piRNA > endo-siRNA.

### 2.10. miRNA Profiling Analysis

Perfectly aligned reads annotating to miRNAs were initially counted; then miRNA expression levels were normalized using log2-RPM within each sample. An RPM value ≥1 for each mature miRNA was regarded as indicating expression. To identify miRNA signatures in mouse SSCs, each mature miRNA with ≥2-fold changes between SSCs and the other four cell types was regarded as an SSC-specific high expression miRNA.

### 2.11. piRNA Profiling Analysis

Because of clustering and repeat-derived characteristics of piRNAs, we analyzed piRNA expression using modified RPM normalization. Briefly, we used weighted #reads, *ω*_piRNA_ = #Reads/#Hits, instead of the number of reads (#Reads) to calculate RPM values. The genome-wide distributions of piRNA expression on both strands were compared among four samples.

### 2.12. Endo-siRNA Analysis

Endo-siRNA was identified on the basis of three stringent screening criteria analogous to those described previously [35]: (1) length of small RNA ranged from 18–23 nt; (2) reads of small RNAs perfectly matched to the mouse genome (mm9); (3) repeat-derived reads. As for piRNA analysis, the weighted expression was calculated and compared for putative endo-siRNA profiles.

## 3. Results

### 3.1. Derivation and Characterization of Mouse SSCs and MSCs

Mouse SSCs were derived and expanded according to the protocol developed by Kanatsu-Shinohara et al. [31]. The cells displayed typical germ stem cell morphology, expressed SSC markers including GFR*α*1, PLZF (ZBTB16), NGN3 (Neurog3), LIN28, and E-cadherin (CDH1) [36] (Supplementary Figure  S1), and could be maintained in culture for more than 30 passages. Mouse MSCs were isolated and cultured as described previously [37]. The identity of the MSCs was verified by their spindle-shaped morphology, the expression of the MSC markers Sca-1 and CD44 and absence of hematopoietic markers CD45 and CD11b (Supplementary Figure  S2), and their abilities to differentiate into adipocytes, osteocytes, and chondrocytes in culture (data not shown) [38].

### 3.2. Overview of Small Noncoding RNA High-Throughput Sequencing

**Figure 1:**
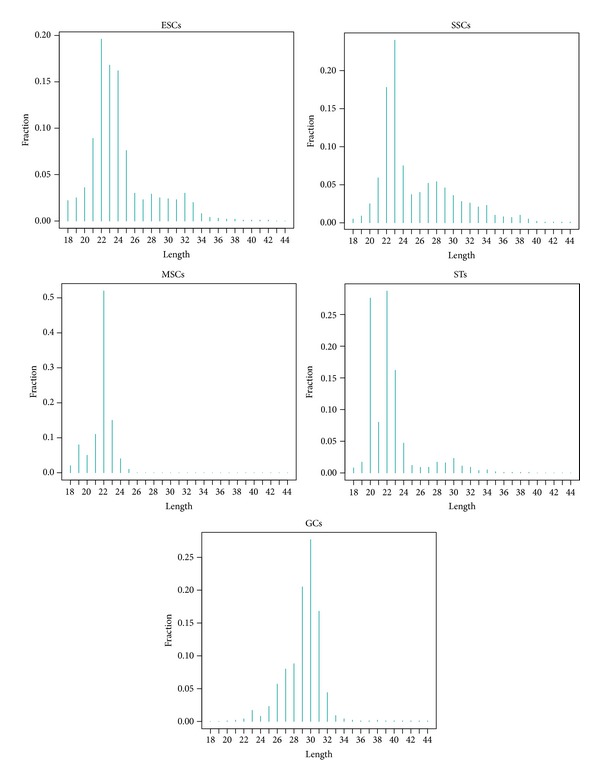
Size distribution of small RNAs in mouse ESCs, SSCs, MSCs, STs, and GCs. Perfectly mapped reads ≥18 nt long were densely plotted for each sample in this study.

**Figure 2:**
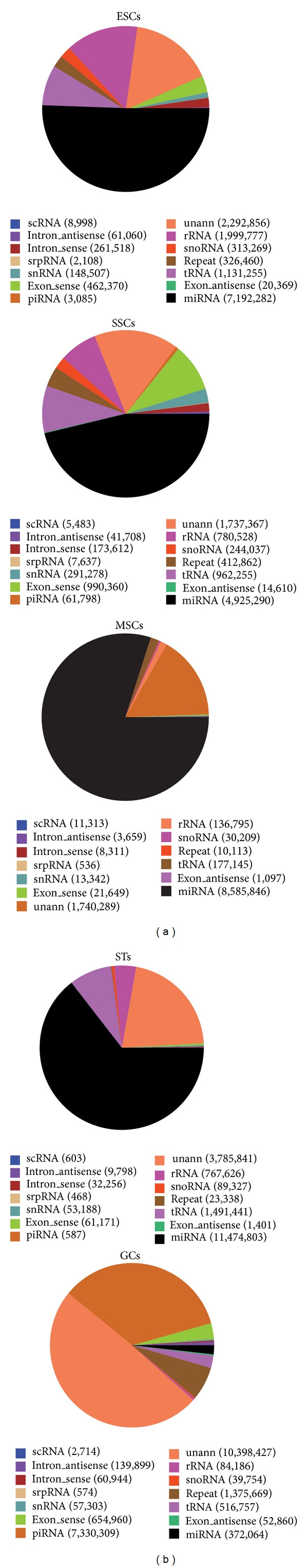
Small RNA annotations for mouse ESCs, SSCs, MSCs, STs, and GCs. The pie chart on the left for each cell type indicates the relative frequency of the annotated noncoding RNAs, and the right panel shows the absolute number of reads annotated.

Small RNAs were separated on 15% denaturing polyacrylamide gels and fragments of 18–40 nt were extracted and purified and used to construct a cDNA library, using the Illumina small RNA sample preparation kit (Illumina, San Diego, CA, USA). Sequencing using an Illumina HiSeq 2000 produced 14.22 × 10^6^, 10.65 × 10^6^, 21.09 × 10^6^, 17.79 × 10^6^, and 10.74 × 10^6^ clean reads from mouse ESCs, SSCs, developing GCs, STs, and MSCs, respectively. Around 75% of the total reads were perfectly matched to the mouse genome using the short oligonucleotide alignment program (SOAP2) [34] (Table 1). The length distributions of the mapped reads were compared among the five samples. Small RNAs in ESCs, SSCs, MSCs, and STs exhibited major length peaks at 22-23 nt, whereas those in GCs displayed a peak at 27–30 nt (Figure 1). The matched small RNA reads in each sample were further annotated and categorized. The major type of annotated small RNA in developing GCs was piRNA, whereas miRNAs accounted for about 60% of total annotated reads in the other four cell samples (Figure 2). This suggests that there is a special requirement for piRNAs in spermatogenesis, as reported by previous studies [39, 40].

### 3.3. miRNA Signature of Mouse SSCs

**Figure 3:**
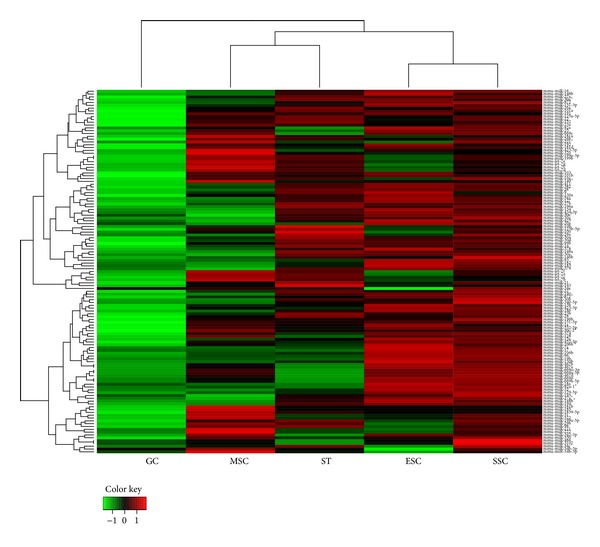
Heat map of five cell types determined by normalized miRNA expression. Each mature miRNA with log2-transformed expression level was used for clustering.

miRNAs represent the most significant class of small RNAs in many key biological processes, including development, cell differentiation, the cell cycle, and apoptosis. To gain further insight into their functional roles in SSCs, we examined the miRNA expression profiles of these samples. After log2-read per million (RPM) normalization, mature miRNAs with RPM ≥ 1 were retained for further analysis. Heat map analysis (Figure 3) showed that SSCs were clustered with ESCs, whereas MSCs were clustered with STs. This clustering pattern was not influenced by the RPM threshold (data not shown), suggesting that SSCs resembled ESCs in terms of miRNA expression. This similarity in miRNA signatures might provide a molecular clue to understanding the spontaneous conversion of SSCs into gPSCs.

We identified a total of 128 mature miRNAs that were highly expressed specifically in mouse SSCs (Table  S3), of which an X-linked miRNA cluster including numerous miRNAs was significantly expressed in SSCs. We also compared our data with previous study (raw data were obtained from Gene Expression Omnibus (GEO; http://www.ncbi.nlm.nih.gov/geo/) with the GSE29613 accession number GSE2564) to confirm the quality of our data. There were high correlations between our data and previous report (data not shown) [26]. Some components of this miRNA cluster (miR-883a, 883b) have previously been reported to be highly or specifically expressed in testes [41]. We also identified 232 miRNAs specifically expressed in ESCs (Table  S4). One of the most notable miRNA features was the miR-302-367 cluster, which has been shown to be specifically expressed in ESCs and to play vital roles in reprogramming somatic cells into pluripotent stem cells [42–47]. Other miRNA clusters (miR-290 cluster, miR-200b-200a-429 cluster, and miR-106a-363 cluster) were also particularly abundant in ESCs compared with SSCs. The functional importance of the miR-290 cluster in stem cell pluripotency has been demonstrated previously [48].

### 3.4. piRNA and Endo-siRNA Profiles

**Figure 4:**
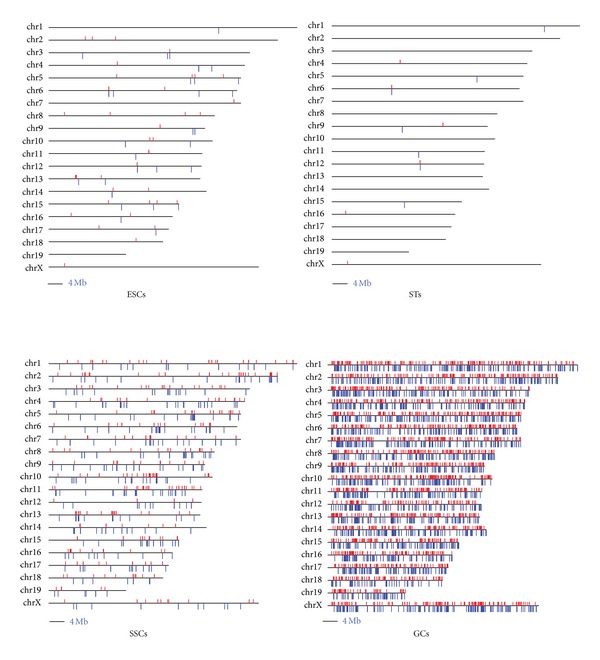
Genome-wide distribution of piRNA expression in four cell types. MSCs were excluded because of their low piRNA expression signal. Red and blue bars represent the plus and minus strands of expressed piRNA, respectively.

**Figure 5:**
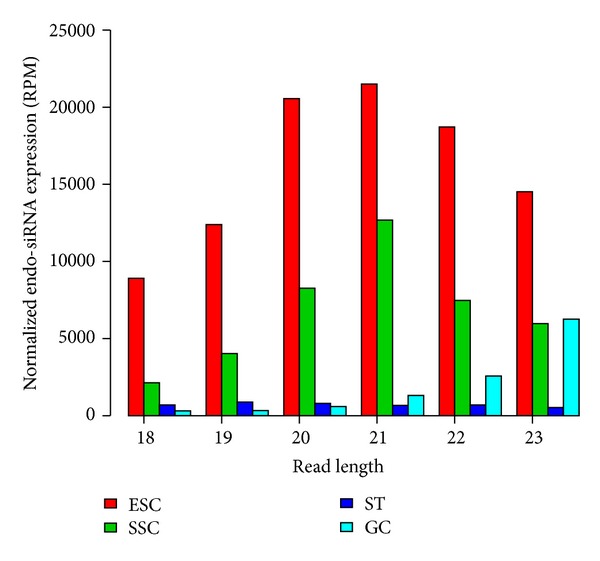
Relative endo-siRNA expression level as a function of read length. TPM denotes the total tag count per 10 million.

piRNAs play important regulatory roles during spermatogenesis [39, 49]. Our genome-wide mapping of piRNAs consistently showed the highest enrichment of piRNA expression in developing GCs, followed by SSCs, ESCs, and STs (Figure 4 and Supplementary Figure  S3). piRNA expression has been reported to cluster on one strand [50]. Because of the repeat-enriched property of piRNAs, we evaluated the frequency of this characteristic in piRNAs. Compared with ESCs and SSCs, endogenous retrovirus 1,2,3-derived repeats for piRNAs were significantly increased in developing GCs (Table  S5), coincident with the suggestion that piRNAs are derived from retrotransposons [51]. We also examined the dynamics of endo-siRNAs during mouse spermatogenesis. Based on stringent criteria, we calculated weighted expression levels of endo-siRNAs and found that ESCs expressed the most abundant endo-siRNAs, followed by SSCs, with the lowest levels in developing GCs (Figure 5). This suggests that the trend for endo-siRNA expression profiles was similar to that for miRNAs.

## 4. Discussion

**Figure 6:**
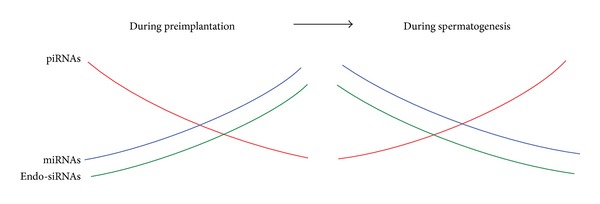
Schematic diagram of small RNA transition during mouse spermatogenesis.

In this study, we used high-throughput sequencing to investigate the small RNA signatures and transitions during mouse spermatogenesis. Overall decreases in miRNAs and endo-siRNAs, in parallel with a gradual increase in piRNAs, were a feature of small RNA transition during mouse spermatogenesis (Figure 6). Although the mechanisms responsible for small RNA transitions remain unclear, these results provide insights into the interactive dynamic gene regulation at the posttranscriptional level during reproduction and development in mice. Moreover, the quantitative regulation by small RNAs further illuminates the spatiotemporal sophistication of gene expression in normal development, implying that spatiotemporal abnormalities of small RNAs may play roles in disease states.

Based on the opposite trends in small RNAs during mouse reproduction and development, focusing on any one type (or class) of small RNAs would only provide information on one aspect of gene regulation, and investigation of small RNAs at the system level is necessary to address posttranscriptional regulation and transition during mouse spermatogenesis in a quantitative manner. Further studies are also needed to determine if the pattern of small RNA transition during mouse spermatogenesis is conserved in humans. These results have potential implications for the reprogramming of SSCs to ESCs based on small RNAs.

The importance of SSCs in spermatogenesis and the ease of reprogramming them into gPSCs [2, 36, 52, 53] suggest that an understanding of the molecular properties of SSCs is essential. As noncanonical regulators of gene expression, small noncoding RNAs have been the subject of intensive studies over the past decade, and their roles in regulating SSC function and spermatogenesis have been investigated [26, 54–57]. In order to identify novel small noncoding RNAs potentially responsible for the functional properties of SSCs, we compared the genome-wide small RNA expression profiles of different mouse testis-derived cell populations, including somatic cells, developing GCs, and in vitro expanded SSCs. We also examined mouse ESCs and MSCs to investigate the similarities between SSCs and ESCs in terms of small noncoding RNA expression profiles. We identified a list of novel miRNAs that were specifically and abundantly expressed in mouse SSCs. Further investigation aimed at the functional dissection of these novel miRNAs could generate new insights into the regulation of SSC activities. Interestingly, our data demonstrated that SSCs displayed similar miRNA expression profiles to ESCs. This similarity was unique to SSCs, and the miRNA signature of MSCs, the other somatic stem-cell type possessing multipotency, did not resemble that of ESCs. Overall, these results indicate that SSCs are primed to become ES-like cells, partially at the miRNA expression level, and this transition can occur easily under suitable culture conditions. miRNAs represent a higher regulatory layer of gene function and cell behavior, and the similarities in miRNA signatures between ESCs and SSCs provide new clues to understanding the molecular basis of the spontaneous reprogramming of unipotent SSCs into multipotent gPSCs. The results of this study may shed light on the mechanisms responsible for determining pluripotency and aid in the development of new ways to treat germline tumors.

## 5. Conclusion

Further investigation of SSC-specific miRNAs's functional annotation will allow insights into the mechanisms involved in the regulation of SSC activities. And the similarity of miRNA signatures between SSCs and ESCs will provide a new clue to understanding the molecular basis underlying the easy conversion of SSCs to ESCs.

## Supplementary Material

Figure S1 and figure S2 indicate the generation and characterization of mouse SSCs. Table S1 is information about antibodies used in this research, and table S2 is polymerase chain reaction primers and conditions for gene expression analysis. In table S3 and table S4, we list the up-regulated miRNAs in mouse SSCs and up-regulated miRNAs in mouse ESCs. Then we summarize the repeat enrichment for piRNAs in mouse ESCs, SSCs and GCs in table S5.

## Figures and Tables

**Table 1:** Summary of small RNA sequencing in mouse ESCs, SSCs, GCs, STs, and MSCs.

	Total clean reads	Mapping to genome	Percentage
ESC	14,223,914	10,477,211	73.66
SSC	10,648,825	8,379,103	78.69
GC	21,086,420	17,136,082	81.27
ST	17,791,848	13,255,304	74.50
MSC	10,740,304	8,832,696	82.24
