# Supplementary material for: miRNA Signature in Mouse Spermatogonial Stem Cells Revealed by High-Throughput Sequencing
Source: Biomed Res Int. 2014 Jul 20;2014:154251. doi: 10.1155/2014/154251 (PMC4124761; doi:10.1155/2014/154251)
Supplement: Supplementary file 1 — Figure S1 and figure S2 indicate the generation and characterization of mouse SSCs. Table S1 is information about antibodies used in this research, and table S2 is polymerase chain reaction primers and conditions for gene expression analysis. In table S3 and table S4, we list the up-regulated miRNAs in mouse SSCs and up-regulated miRNAs in mouse ESCs. Then we summarize the repeat enrichment for piRNAs in mouse ESCs, SSCs and GCs in table S5. [file 154251.f1.pdf]

**Table S1. Antibodies used in this research**

| Antibody against | Antibody type | Dilution | Source<br>catalog number |
|---|---|---|---|
| GFR α1 | Rabbit polyclonal antibody IgG | 1: 200 | Abcam (Cambridge, MA)<br>ab8026 |
| Texas Red (TR)-conjugated goat anti-rabbit IgG | Goat polyclonal antibody IgG | 1:100 | Santa Cruz Biotechnologies<br>sc-2780 |
| Anti-Mouse MHC Class II (I-A/I-E) PE | Rat monoclonal antibody IgG2b | 1:9 | eBioscience<br>12-5321-81 |
| Anti-Mouse CD45 PE | Rat monoclonal antibody IgG2b | 1:9 | eBioscience<br>12-0451-81 |
| Anti-Mouse CD11b PE | Rat monoclonal antibody IgG2b | 1:9 | eBioscience<br>12-0112-81 |
| Anti-mouse/human CD44 PE | Rat monoclonal antibody IgG2b | 1:9 | Biolegend<br>103007 |
| Rat IgG2b K Isotype Control PE | Rat monoclonal antibody IgG2b | 1:9 | eBioscience<br>12-4031-81 |
| Anti-Mouse MHC Class I (H-2Db) PE | Mouse monoclonal antibody IgG2a | 1:9 | eBioscience<br>12-5999-81 |
| Mouse IgG2a K Isotype Control PE | Mouse monoclonal antibody IgG2a | 1:9 | eBioscience<br>12-4724-41 |

| | | | |
|---|---|---|---|
| Anti-Mouse Ly-6A/E (Sca-1) PE | Rat monoclonal antibody IgG2a | 1:9 | eBioscience 12-5981-81 |
| Rat IgG2a K Isotype Control PE | Rat monoclonal antibody IgG2a | 1:9 | eBioscience 12-4321-41 |

**Table S2. PCR primers and conditions for gene expression analysis**

| Genes | Primer sequences (5'-3', Forward and Reverse) | Annealing temp | Length (bp) | Accession No. |
|---|---|---|---|---|
| GFRa1 | 5'TGGACAGGCAGGATGAAATAGAGC3'<br>5' TACTTGTGCCATTCAGTTCACCTTC 3' | 56 | 421 | NM_010279 |
| PLZF | 5'CCATACTGGCACGGACAT3'<br>5' CTGTAGTGCGTCTCCAAC 3' | 56 | 336 | NM_001033324 |
| E-cadherin | 5'GGCATTCCTTGTTCGGCTAT3'<br>5'AAAACATTGGTTGAGATAAG3' | 56 | 394 | NM_009864 |
| NGN3 | 5'CTTCGCCCACAACTACATCT3'<br>5'TTTCCACTAGCACCCACCAC3' | 56 | 308 | NM_009719 |
| LIN28 | 5'GGGACTCAACTCCTTAGCCT3'<br>5'AACAGGTAAGGATAAGCCAT3' | 56 | 542 | NM_145833 |
| Oct4 | 5'GTATTCCCAACGAGAAGAGT3'<br>5'ATGATGAGTGACAGACAGGC3' | 57 | 394 | NM_013633 |
| 18SRNA | 5' GGTTCCTTTGGTCGCTCGCT3'<br>5'GCTGCCTTCCTTGGATGTGG3' | 58 | 364 | NM_145833 |

## Table S3. miRNAs highly expressed in mouse SSCs

| miRNAs | ESCs | SSCs | MSCs | STs | GCs |
|---|---|---|---|---|---|
| mmu-let-7g* | 0.141 | 0.47 | 0 | 0.169 | 0 |
| mmu-miR-101b* | 1.265 | 5.447 | 0.093 | 0.169 | 0.142 |
| mmu-miR-10a | 589.36 | 8229.64 | 3607.16 | 3293.475 | 77.585 |
| mmu-miR-1195 | 7.241 | 31.928 | 0.093 | 0 | 0.142 |
| mmu-miR-129-2-3p | 6.468 | 28.454 | 12.011 | 1.742 | 0.142 |
| mmu-miR-146b | 772.642 | 8203.159 | 303.343 | 1650.138 | 114.671 |
| mmu-miR-153 | 0.633 | 1.315 | 0 | 0.225 | 0.047 |
| mmu-miR-155 | 26.083 | 164.431 | 3.445 | 55.419 | 0.854 |
| mmu-miR-155* | 0 | 1.503 | 0 | 0.112 | 0 |
| mmu-miR-184 | 134 | 537.242 | 0.652 | 20.684 | 226.212 |
| mmu-miR-190* | 0.07 | 0.282 | 0 | 0 | 0 |
| mmu-miR-190b | 1.898 | 5.447 | 0.931 | 0.843 | 0.427 |
| mmu-miR-1930 | 0.07 | 0.376 | 0.093 | 0 | 0.047 |
| mmu-miR-195 | 92.239 | 456.294 | 1.583 | 46.482 | 8.631 |
| mmu-miR-195* | 0.562 | 12.396 | 0.186 | 0.393 | 0.047 |
| mmu-miR-196b | 70.374 | 318.908 | 64.337 | 63.006 | 3.367 |
| mmu-miR-201* | 0 | 0.094 | 0 | 0 | 0 |
| mmu-miR-203 | 173.792 | 1063.779 | 0.186 | 60.983 | 5.975 |
| mmu-miR-203* | 0.352 | 3.756 | 0 | 0 | 0.047 |
| mmu-miR-215 | 3.234 | 31.271 | 1.862 | 4.215 | 0.285 |
| mmu-miR-216b | 0 | 1.127 | 0.186 | 0 | 0 |
| mmu-miR-222* | 4.078 | 21.974 | 10.428 | 5.621 | 0.142 |
| mmu-miR-224 | 14.623 | 72.872 | 20.763 | 14.726 | 0.379 |
| mmu-miR-26b* | 0.773 | 2.817 | 0 | 0.281 | 0.047 |
| mmu-miR-297a* | 10.616 | 27.233 | 1.024 | 0 | 0.047 |
| mmu-miR-297a-5* | 1.758 | 5.447 | 0 | 0 | 0.142 |
| mmu-miR-297b-3p | 10.616 | 27.233 | 5.307 | 0 | 0.047 |
| mmu-miR-297c | 13.85 | 30.426 | 5.307 | 0.056 | 0.095 |
| mmu-miR-297c* | 10.616 | 27.233 | 0.279 | 0 | 0.047 |
| mmu-miR-29c* | 0.352 | 2.066 | 0.652 | 0.731 | 0.047 |
| mmu-miR-3065 | 0 | 0.376 | 0 | 0.112 | 0 |
| mmu-miR-3065* | 0 | 0.188 | 0 | 0.056 | 0 |
| mmu-miR-3082-5p | 0 | 0.094 | 0 | 0 | 0 |
| mmu-miR-3083 | 0 | 0.47 | 0 | 0.112 | 0 |
| mmu-miR-3084* | 1.687 | 5.541 | 0.186 | 1.574 | 0.142 |
| mmu-miR-3086-3p | 0 | 0.47 | 0.093 | 0 | 0 |
| mmu-miR-3096-3p | 2.039 | 10.987 | 1.49 | 0.337 | 0.664 |
| mmu-miR-3096b-3p | 1.969 | 10.705 | 0 | 0.337 | 0.664 |
| mmu-miR-30b | 66.297 | 356.471 | 10.521 | 103.137 | 7.967 |

| | | | | | |
|---|---|---|---|---|---|
| mmu-miR-30c | 274.397 | 760.741 | 20.949 | 252.588 | 22.621 |
| mmu-miR-31* | 4.921 | 46.86 | 11.08 | 0.955 | 0.427 |
| mmu-miR-3102-3p.2 | 0 | 0.282 | 0.093 | 0.112 | 0 |
| mmu-miR-3105-3p | 0.07 | 0.376 | 0 | 0 | 0 |
| mmu-miR-3105-5p | 0.211 | 0.657 | 0 | 0 | 0 |
| mmu-miR-3107 | 5.554 | 47.799 | 5.586 | 1.63 | 2.988 |
| mmu-miR-3107* | 0.07 | 4.695 | 0 | 0 | 0.095 |
| mmu-miR-339-5p | 9.843 | 26.951 | 2.235 | 1.855 | 0.427 |
| mmu-miR-340-3p | 4.71 | 24.698 | 0 | 3.934 | 0.427 |
| mmu-miR-340-5p | 70.374 | 290.642 | 15.083 | 49.124 | 11.809 |
| mmu-miR-344 | 5.765 | 13.992 | 6.331 | 2.642 | 0.047 |
| mmu-miR-344d | 0.844 | 1.784 | 0.466 | 0.506 | 0.047 |
| mmu-miR-346 | 0.281 | 2.348 | 0 | 0 | 0 |
| mmu-miR-346* | 0 | 0.094 | 0 | 0 | 0 |
| mmu-miR-3470a | 19.755 | 92.686 | 0.652 | 2.136 | 6.45 |
| mmu-miR-3470b | 25.309 | 96.067 | 0.745 | 2.023 | 12.188 |
| mmu-miR-350* | 0.773 | 2.535 | 0.372 | 0.169 | 0 |
| mmu-miR-3572 | 0 | 0.188 | 0 | 0.056 | 0 |
| mmu-miR-377 | 7.944 | 16.152 | 0.838 | 4.215 | 0 |
| mmu-miR-463 | 0.844 | 958.697 | 0 | 0.112 | 7.256 |
| mmu-miR-463* | 0.422 | 457.327 | 0 | 0 | 2.419 |
| mmu-miR-465a-3p | 71.288 | 26396.152 | 0.093 | 2.304 | 178.741 |
| mmu-miR-465a-5p | 9.561 | 7398.469 | 0 | 0.45 | 64.212 |
| mmu-miR-465b-3p | 71.218 | 26325.346 | 0.093 | 2.248 | 174.141 |
| mmu-miR-465c-3p | 70.867 | 26171.15 | 0.093 | 2.248 | 173.287 |
| mmu-miR-465c-5p | 15.889 | 8864.922 | 0 | 0.843 | 55.249 |
| mmu-miR-466a-3p | 280.232 | 611.992 | 14.339 | 0.843 | 3.794 |
| mmu-miR-466b-3p | 297.176 | 640.54 | 14.711 | 0.955 | 3.984 |
| mmu-miR-466c-3p | 297.176 | 640.54 | 14.711 | 0.955 | 3.984 |
| mmu-miR-466e-3p | 297.316 | 640.634 | 14.804 | 0.955 | 3.984 |
| mmu-miR-466j | 0.07 | 0.657 | 0 | 0 | 0 |
| mmu-miR-466p-3p | 296.191 | 640.165 | 14.618 | 0.955 | 3.984 |
| mmu-miR-466q | 0 | 0.094 | 0 | 0 | 0 |
| mmu-miR-467e | 42.534 | 103.58 | 19.459 | 0.45 | 0.854 |
| mmu-miR-470 | 28.122 | 19619.16 | 0 | 2.698 | 96.46 |
| mmu-miR-470* | 0.07 | 115.787 | 0 | 0 | 0.996 |
| mmu-miR-471-3p | 0 | 83.108 | 0 | 0 | 0.664 |
| mmu-miR-471-5p | 0.281 | 206.22 | 0 | 0.056 | 2.703 |
| mmu-miR-483 | 2.953 | 14.18 | 0 | 0 | 0.047 |
| mmu-miR-483* | 0.281 | 3.475 | 0 | 0 | 0.047 |
| mmu-miR-486 | 5.695 | 47.611 | 5.586 | 1.63 | 3.035 |

| | | | | | |
|---|---|---|---|---|---|
| mmu-miR-486* | 0.422 | 21.786 | 1.583 | 0.562 | 0.19 |
| mmu-miR-497 | 13.217 | 38.408 | 4.376 | 5.789 | 0.664 |
| mmu-miR-497* | 0.562 | 1.503 | 0 | 0.056 | 0.047 |
| mmu-miR-499* | 0 | 0.094 | 0 | 0 | 0 |
| mmu-miR-5103 | 0.07 | 0.282 | 0 | 0 | 0 |
| mmu-miR-5106 | 0 | 0.188 | 0 | 0 | 0 |
| mmu-miR-511-3p | 1.898 | 24.979 | 0 | 10.735 | 0.19 |
| mmu-miR-5121 | 1.898 | 5.071 | 0 | 0.112 | 0 |
| mmu-miR-547 | 0 | 0.282 | 0 | 0 | 0.047 |
| mmu-miR-592 | 0.422 | 2.442 | 0 | 0 | 0 |
| mmu-miR-664 | 0.703 | 7.7 | 1.49 | 3.71 | 0.047 |
| mmu-miR-668 | 2.039 | 4.226 | 0.093 | 1.967 | 0 |
| mmu-miR-669c* | 0.211 | 0.563 | 0.093 | 0 | 0 |
| mmu-miR-669i | 0 | 0.188 | 0 | 0 | 0 |
| mmu-miR-669m-3p | 1.476 | 3.568 | 0.466 | 0 | 0 |
| mmu-miR-671-5p | 18.701 | 43.009 | 0.745 | 16.187 | 0.379 |
| mmu-miR-674 | 13.92 | 32.304 | 5.866 | 3.991 | 0.379 |
| mmu-miR-678 | 0 | 0.094 | 0 | 0 | 0 |
| mmu-miR-680 | 0 | 0.094 | 0 | 0 | 0 |
| mmu-miR-682 | 0.07 | 0.188 | 0 | 0 | 0 |
| mmu-miR-684 | 0 | 0.188 | 0 | 0 | 0 |
| mmu-miR-692 | 0.141 | 1.784 | 0 | 0 | 0.047 |
| mmu-miR-741 | 16.381 | 15663.888 | 0 | 5.058 | 138.335 |
| mmu-miR-741* | 0.562 | 154.008 | 0 | 0 | 1.612 |
| mmu-miR-742 | 0 | 24.51 | 0 | 0 | 0.332 |
| mmu-miR-742* | 0 | 11.457 | 0 | 0 | 0 |
| mmu-miR-743a | 3.867 | 8072.909 | 0 | 1.237 | 60.276 |
| mmu-miR-743a* | 0.352 | 223.311 | 0 | 0 | 1.328 |
| mmu-miR-743b-3p | 1.969 | 1840.954 | 0 | 0.393 | 20.867 |
| mmu-miR-743b-5p | 0.07 | 77.379 | 0 | 0.056 | 0.427 |
| mmu-miR-871-3p | 62.43 | 47916.648 | 0 | 7.194 | 296.589 |
| mmu-miR-871-5p | 0.844 | 1991.769 | 0 | 0.056 | 14.607 |
| mmu-miR-872* | 124.016 | 277.683 | 14.99 | 28.777 | 1.66 |
| mmu-miR-873 | 0.07 | 4.508 | 0.186 | 0.056 | 0.047 |
| mmu-miR-873* | 0 | 0.094 | 0 | 0 | 0 |
| mmu-miR-875-5p | 0 | 0.094 | 0 | 0 | 0 |
| mmu-miR-878-3p | 2.109 | 1298.547 | 0 | 0.169 | 7.351 |
| mmu-miR-878-5p | 83.803 | 20216.409 | 0 | 5.002 | 137.15 |
| mmu-miR-879 | 0.352 | 0.939 | 0 | 0.056 | 0.047 |
| mmu-miR-879* | 0 | 0.188 | 0 | 0 | 0 |

| | | | | | |
|---|---|---|---|---|---|
| mmu-miR-880 | 17.646 | 9533.634 | 0 | 1.405 | 60.94 |
| mmu-miR-880* | 0 | 0.094 | 0 | 0 | 0 |
| mmu-miR-881 | 8.507 | 5107.887 | 0 | 1.405 | 52.403 |
| mmu-miR-881* | 0.422 | 236.646 | 0 | 0.056 | 1.802 |
| mmu-miR-883a-3p | 0.211 | 424.46 | 0 | 0 | 3.557 |
| mmu-miR-883a-5p | 0.07 | 141.424 | 0 | 0 | 0.854 |
| mmu-miR-883b-3p | 0 | 100.574 | 0 | 0 | 0.569 |
| mmu-miR-883b-5p | 0.07 | 240.684 | 0 | 0 | 1.565 |

**Table S5. endogenous retrovirus 1,2,3-derived repeats for piRNAs increased in developing GCs**

| ESC | |
|---|---|
| **Repeats** | **Relative Frequency %** |
| DNAtransposon | 9.62 |
| ERV3 | 0.34 |
| Pseudogene | 86.63 |
| SINE1/7SL | 7.17 |
| rRNA | 0.72 |
| tRNA | 5.05 |

| SSC | |
|---|---|
| **Repeats** | **Relative Frequency %** |
| CR1 | 15.61 |
| DNAtransposon | 2.06 |
| ERV1 | 1.07 |
| ERV2 | 1.88 |
| ERV3 | 26.26 |
| L1 | 2.27 |
| Mariner/Tc1 | 0.18 |
| Pseudogene | 22.09 |
| RMER1A | 0.01 |
| RTEX | 0.72 |
| SINE | 7.04 |
| SINE1/7SL | 8.09 |
| SINE2/tRNA | 8.75 |
| Satellite | 0.04 |
| SimpleRepeat | 0.61 |
| TransposableElement | 0.04 |
| hAT | 2.50 |
| rRNA | 0.04 |
| snRNA | 0.29 |
| tRNA | 0.45 |

| GCs | |
|---|---|
| **Repeats** | **Relative Frequency %** |
| CR1 | 8.88 |
| DNAtransposon | 1.08 |
| ERV1 | 0.73 |
| ERV2 | 2.72 |
| ERV3 | 48.60 |
| Gypsy | 0.00 |
| L1 | 2.56 |
| Mariner/Tc1 | 0.17 |
| Pseudogene | 0.00 |
| RMER1A | 0.00 |
| RTEX | 1.74 |
| SINE | 9.91 |
| SINE1/7SL | 9.50 |
| SINE2/tRNA | 9.25 |
| Satellite | 0.04 |
| SimpleRepeat | 0.72 |
| TransposableElement | 0.01 |
| hAT | 3.71 |
| rRNA | 0.01 |
| snRNA | 0.37 |

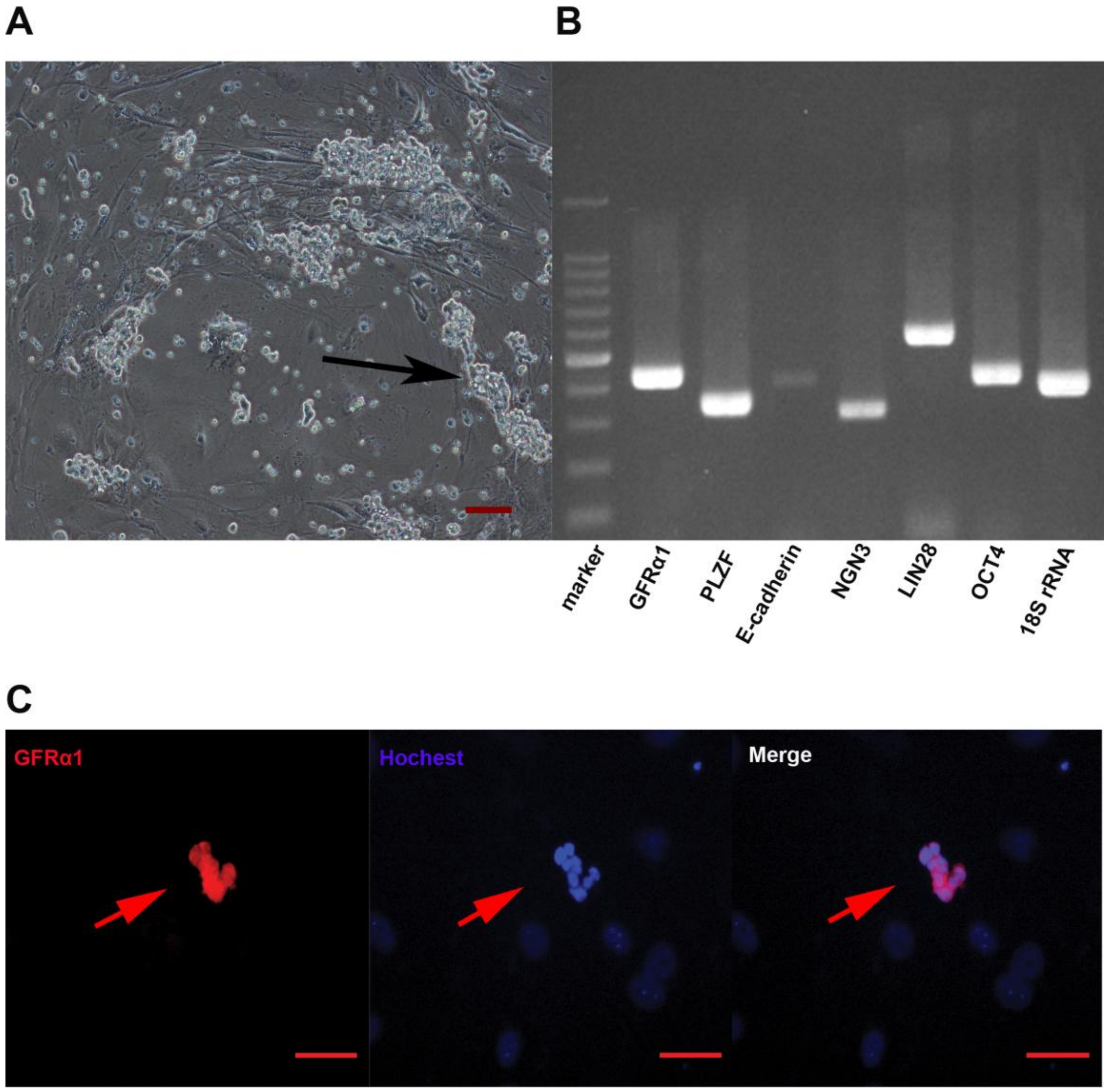
A
B
marker
GFRα1
PLZF
E-cadherin
NGN3
LIN28
OCT4
18S rRNA
C
GFRα1
Hochest
Merge

Figure S1

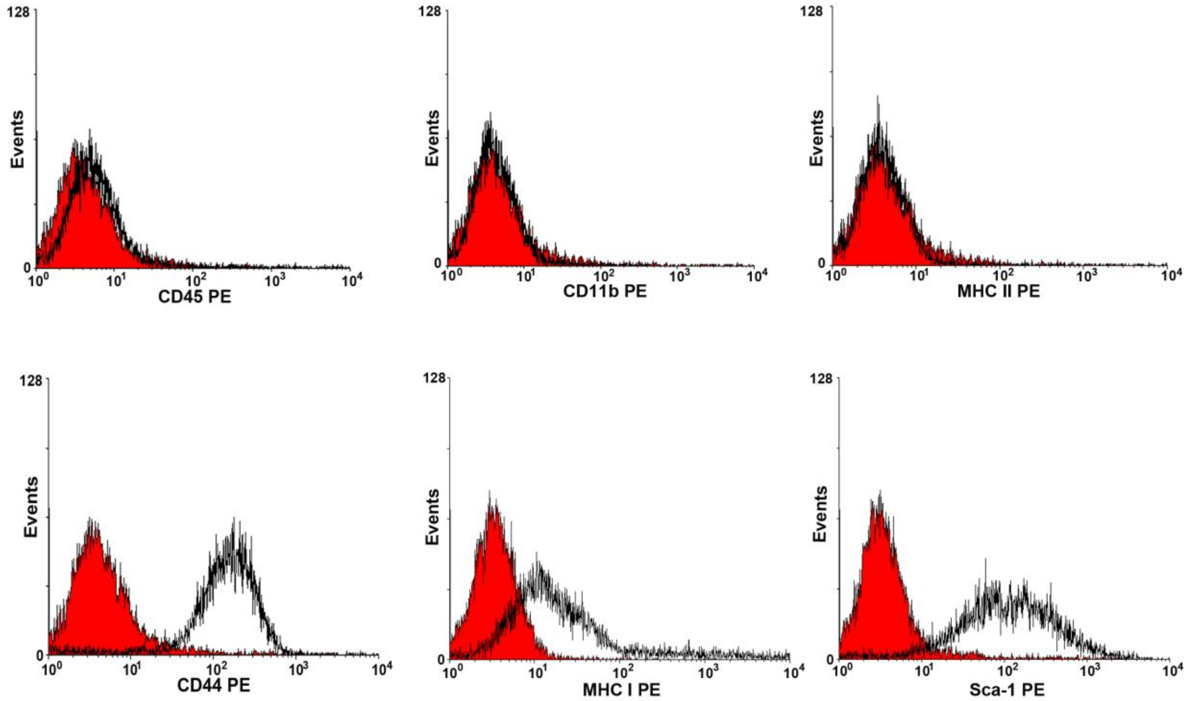


Figure S2

Figure S3

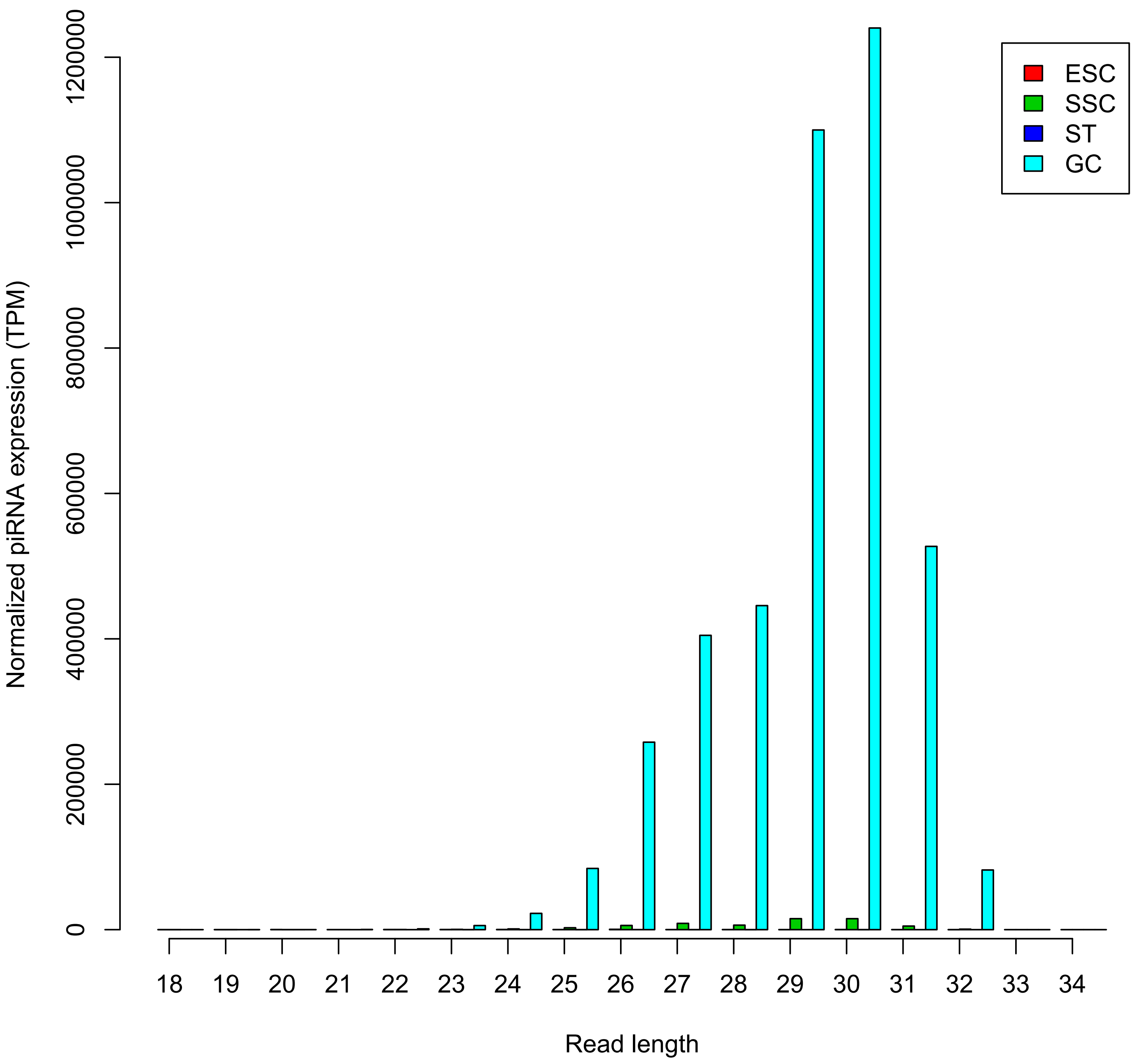

ESC
SSC
ST
GC
Normalized piRNA expression (TPM)
0
200000
400000
600000
800000
1000000
1200000
18
19
20
21
22
23
24
25
26
27
28
29
30
31
32
33
34
Read length
